# Evaluation of Normalization Approaches for Quantitative Analysis of Bile Acids in Human Feces

**DOI:** 10.3390/metabo12080723

**Published:** 2022-08-05

**Authors:** Hans-Frieder Schött, Esther W. L. Chua, Sartaj Ahmad Mir, Bo Burla, Anne K. Bendt, Markus R. Wenk

**Affiliations:** 1 Singapore Lipidomics Incubator, Life Sciences Institute, National University of Singapore, Singapore 117456, Singapore; 2 Department of Biochemistry, Yong Loo Lin School of Medicine, National University of Singapore, Singapore 117596, Singapore; 3 Precision Medicine Translational Research Program, Yong Loo Lin School of Medicine, National University of Singapore, Singapore 117596, Singapore

**Keywords:** bile acids, human feces, human stool, normalization, wet weight, dry weight, protein content, fecal lipids, lipids

## Abstract

Quantitative analysis of bile acids in human feces can potentially help to better understand the influence of the gut microbiome and diet on human health. Feces is a highly heterogeneous sample matrix, mainly consisting of water and indigestible solid material (as plant fibers) that show high inter-individual variability. To compare bile acid concentrations among different individuals, a reliable normalization approach is needed. Here, we compared the impact of three normalization approaches, namely sample wet weight, dry weight, and protein concentration, on the absolute concentrations of fecal bile acids. Bile acid concentrations were determined in 70 feces samples from healthy humans. Our data show that bile acid concentrations normalized by the three different approaches are substantially different for each individual sample. Fecal bile acid concentrations normalized by wet weight show the narrowest distribution. Therefore, our analysis will provide the basis for the selection of a suitable normalization approach for the quantitative analysis of bile acids in feces.

## 1. Introduction

Recent studies have highlighted the influence of diet on the composition of the gut microbiome [1]. Major attempts have been made to identify the composition of a healthy diet and additionally to associate health benefits with the gut microbiome composition [2,3,4]. Absorption and excretion studies have been conducted to investigate the influence of dietary modification on human metabolism [5]. The influences of the gut microbiota on host lipid metabolism are mediated through metabolites produced by the gut microbiota such as short-chain fatty acids, bile acids, and other pro-inflammatory intestinal bacteria-derived factors. Studies focusing on bile acid concentrations in human feces require their accurate quantitation. It is essential for the comparison of data from different samples to normalize bile acid concentrations to a common basis that is reproducible in every sample.

Mass spectrometry-based analysis of clinically relevant analytes depends on the usage of comparable sample amounts to obtain similar matrix conditions in each analyzed sample [6]. Normalization of blood plasma or serum samples is easily feasible by volume. However, identification of the most suitable normalization approaches for advanced analytical techniques which are used in complex biological matrices, such as feces, urine, or tissue samples is challenging. For instance, in urine analysis, several different approaches such as volume, levels of creatinine or cystatin C, or osmolarity have been evaluated for normalization [7,8,9,10,11].

**Figure 1:**
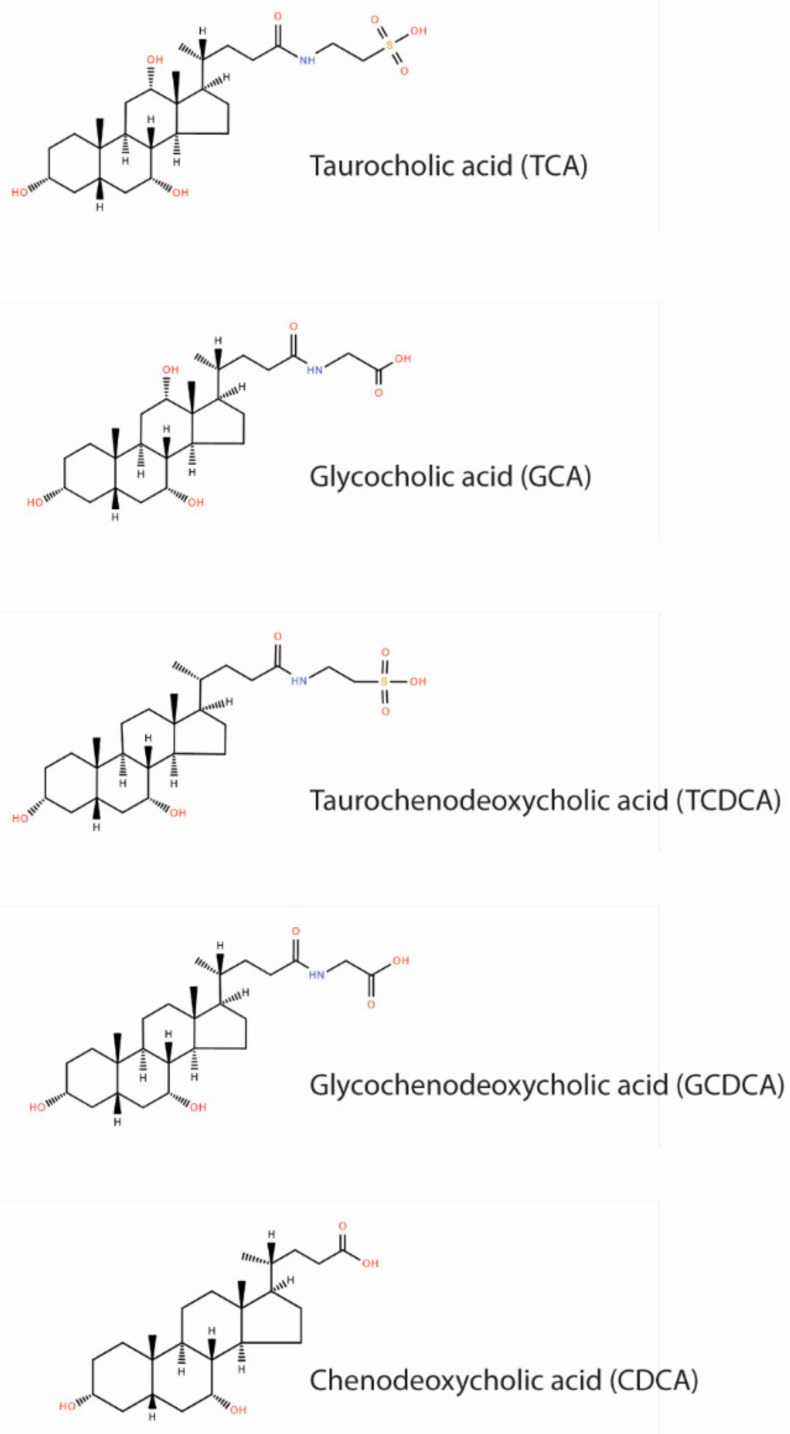
Chemical structure of included bile acids.

The heterogenous nature of feces represents a unique analytical challenge. Until today, no optimal normalization approach has been evaluated and reported [12]. For this reason, we evaluated three different approaches for feces normalization. The concentrations of five bile acids, namely, taurocholic acid (TCA), glycocholic acid (GCA), chenodeoxycholic acid (CDCA), glycochenodeoxycholic acid (GCDCA), and taurochenodeoxycholic acid (TCDCA), were analyzed in 70 human feces samples. The chemical structures of these bile acids are shown in Figure 1.

These bile acids have been selected due to their (i) good detectability in feces and (ii) representation of diverse chemical properties. Taurine conjugated bile acids possess a lower pK_a_ value and show preferred deprotonated carboxyl functions as free or glycine conjugated bile acids [13,14]. Further, the selected bile acids have different water solubilities and show structural diversity due to their hydroxylation and conjugation patterns. This makes the selected bile acids suitable candidates for the evaluation of an improved normalization approach, applicable to a range of bile acids. The concentrations of the five bile acids were normalized by three different approaches, namely, feces sample wet weight, dry weight, and protein content. The aim of this evaluation was to identify a normalization approach that results in the narrowest distribution of measured concentrations, for a more precise detection of significant biological differences in bile acid concentrations.

**Figure 2:**
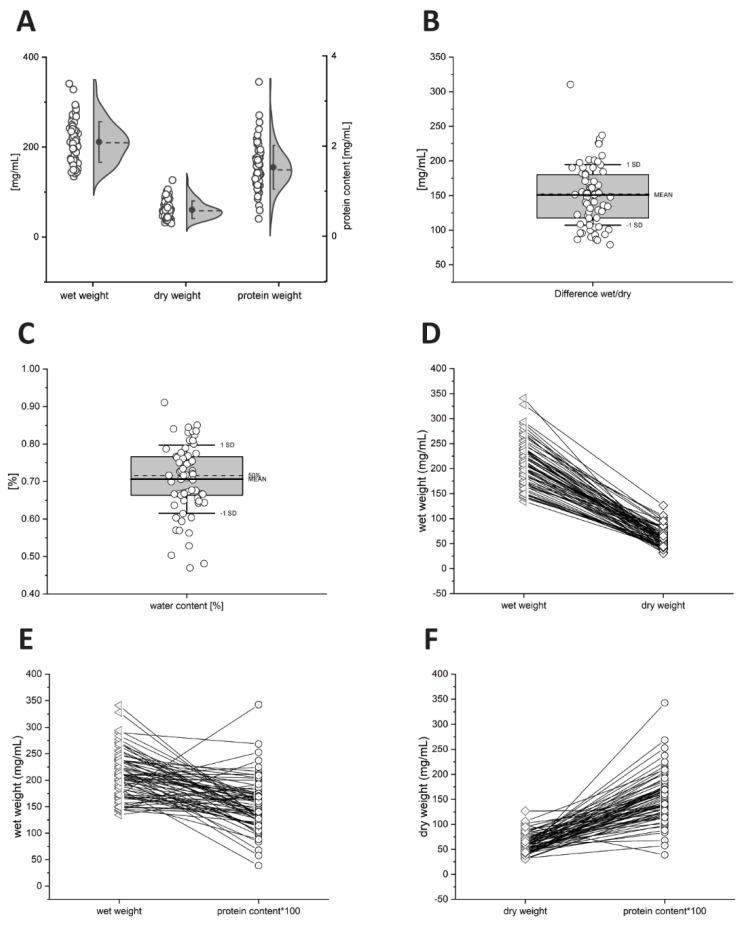
Weight distribution of 70 human feces samples from 42 healthy volunteers. Distribution of wet weight, dry weight, and protein content (**A**), difference of wet weight and dry weight (**B**), water content in percentage (**C**). Correlations of wet and dry weight (**D**), correlations of wet and protein content (**E**), correlations of dry, and protein content (**F**).

## 2. Results

### 2.1. Differences in Wet Weight, Dry Weight and Protein Content of Faeces Samples

The total weight of the 70 human feces samples ranged from 134.6–340.9 mg/mL (wet), 30.5–126.5 mg/mL (dry) and 0.39–3.42 mg/mL (protein) (Figure 2A). The difference between wet weight compared to dry weight ranged from 78.8–310.4 mg/mL, which points to a water content of 47–91% in these feces samples (Figure 2B). Wet and dry weight showed a weak but significant correlation (R = 0.282, *p* = 0.018).

**Figure 3:**
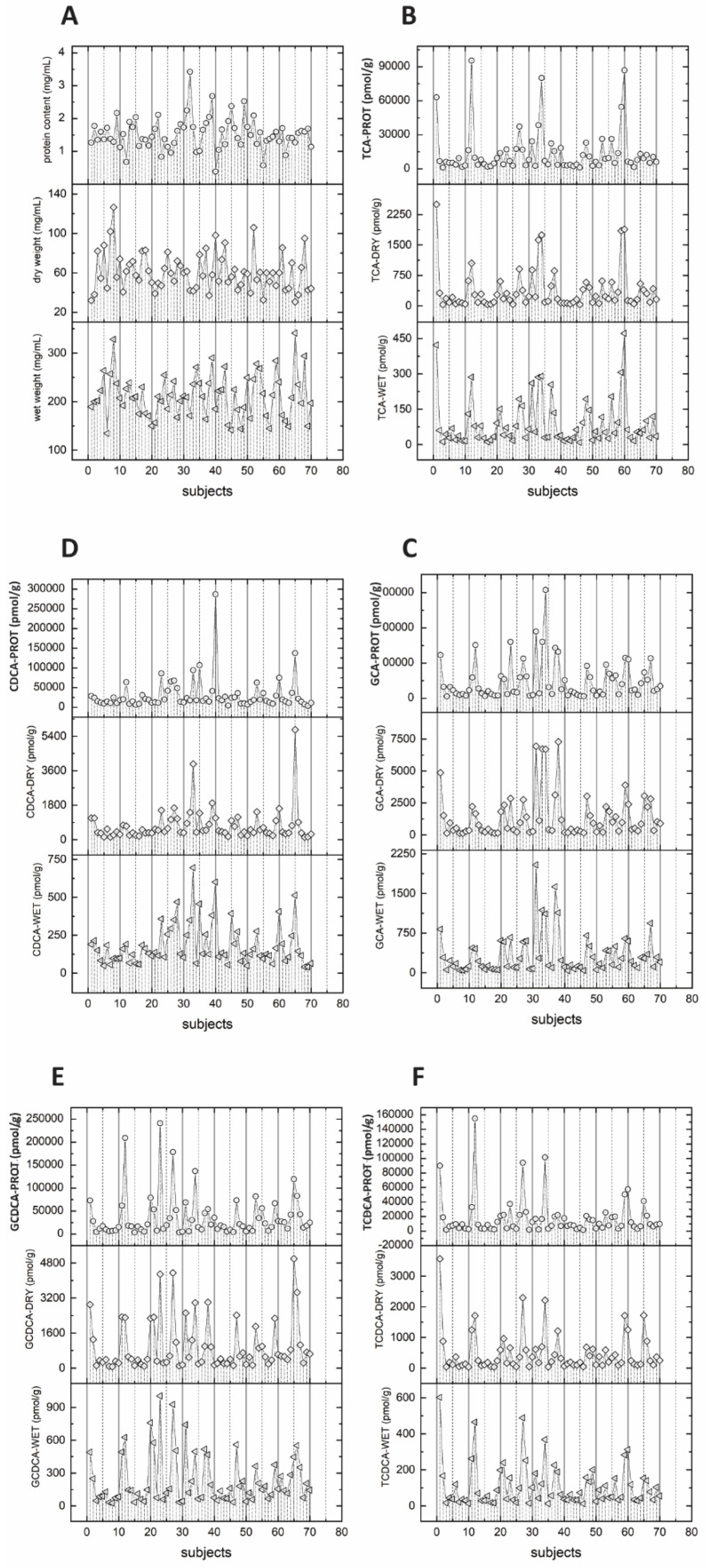
(**A**) Individual wet weight, dry weight, and protein content of 70 human feces samples from 42 volunteers. Distribution of measured concentration of five bile acids, TCA (**B**), GCA (**C**), CDCA (**D**), GCDCA (**E**), and TCDCA (**F**).

Figure 3A shows the characteristics of wet, dry weight, and protein weight for individual samples. Some feces samples showed a high wet weight but a low dry weight and vice versa. This leads to a quite heterogenous relation between wet and dry weight and no common trend is observed. In contrast, protein weight revealed no significant correlations with wet weight (R = −0.061, *p* = 0.617) nor with dry weight (R = −0.080, *p* = 0.511) (Figure 2). For protein weight, an even higher heterogeneity in relation to wet and dry weight was observed. In summary, each sample has an individual association of wet weight, dry weight, and protein weight (Figure 2D–F and Figure 3A).

### 2.2. Differences in Bile Acid Concentrations Based on Normalization Method

Protein weight normalized bile acid concentrations showed the highest nominal value followed by dry weight and wet weight normalized concentrations (Supplementary Figure S1 and Table 1). Bile acid concentrations obtained by all three different approaches showed strong correlations (see Table 2). However, the different normalization approaches resulted in variations in the concentration of individual bile acids. These concentrations can show a different interval for individual concentrations to the mean bile acid concentration in the sample. Normalization approaches influence this interval of bile acid concentrations for individual samples compared to the total bile acid concentrations (see Figure 3B–F). For all five bile acids, normalized by the three different approaches, several statistical parameters were calculated including mean, standard deviation, coefficient of variation (%CV), standard error of the mean, mean absolute deviation (in percent), median, median absolute deviation, median absolute deviation (in percent), range, and range (in percentage) (see Table 1).

Taurocholic acid shows the lowest coefficient of variation for wet weight normalized concentrations (total 109.2%; female (F) 109.5%; male (M) 110.5%), followed by dry weight normalized (total 132.2%; F 139.5%; M 123.4%) and protein content normalized (total 137.2%; F 144.3%; M 127.5%) bile acid concentrations. Chenodeoxycholic acid shows the lowest coefficient of variation for wet weight normalized concentrations (total 75.4%; F 75.2%; M 76.8%), followed by dry weight normalized (total 115.3%; F 98.7%; M 129.8%) and protein content normalized (total 131.1%; F 91.2%; M 156.5%) bile acid concentrations. Glycochenodeoxycholic acid shows the lowest coefficient of variation for wet weight normalized concentrations (total 97.9%; F 110.6%; M 73.9%), followed by dry weight normalized (total 117.2%; F 120.3%; M 114.9%) and protein content normalized (total 125.7%; F 142.0%; M 85.1%) bile acid concentrations. Taurochenodeoxycholic acid shows the lowest coefficient of variation for wet weight normalized concentrations (total 107.2%; F 117.5%; M 80.3%), followed by dry weight normalized (total 133.1%; F 143.5%; M 108.3%) and protein content normalized (total 145.4%; F 157.7%; M 108.3%) bile acid concentrations. In contrast to the above-mentioned four bile acids, glycocholic acid showed the lowest coefficient of variation for protein content normalized concentrations for all samples and the subgroups female and male participants (total 111.7%; F 129.6%, M 84.9%), followed by wet weight normalized (total 112.7%; F 123.1%; M 102.2%) and dry weight normalized (total 122.8%; 135.5%; 108.4%) bile acid concentrations. Normalization of bile acid concentrations by wet weight revealed the narrowest distribution of values indicated by the coefficient of variation of the four evaluated bile acids. This result is confirmed when also the robust coefficient of variation, standard error of the mean (in percentage), median absolute deviation (in percent), and range (in percentage) are analyzed for the wet weight. Normalization by dry weight showed a substantial broader distribution of bile acid concentrations. Protein content showed in most cases the widest distribution, except for glycocholic acid.

**Table 1:** The statistical analysis of the wet, dry weight and protein content of the 70 samples from 42 healthy volunteers as well as the measured concentrations of the five bile acids TCA, GCA, CDCA, GCDCA, and TCDCA. Shown are mean (pmol/g), standard deviation (pmol/g), coefficient of variation (in percentage %), robust coefficient of variation (in percentage %), standard error of the mean (pmol/g), standard error of the mean (in percentage %), minimum (pmol/g), median (pmol/g), maximum (pmol/g), range (pmol/g), range in percent of the mean.

	Group	N	Mean	STDEV	CV [%]	Robust CV [%]	SEM	SEM [%]	Minimum	Median	Maximum	Range (Max–Min)	Range/% of mean
wet weight	all	70	211.5	45.1	21.3%	21.1%	5.4	2.5%	134.6	209.6	340.9	206.3	97.6%
	F	36	211.0	38.1	18.0%	18.0%	6.3	3.0%	134.6	209.6	328.2	193.6	91.7%
	M	34	212.0	52.1	24.6%	27.6%	8.9	4.2%	141.4	210.8	340.9	199.6	94.1%
dry weight	all	70	60.6	19.4	32.1%	33.6%	2.3	3.8%	30.5	58.5	126.5	96.0	158.4%
	F	36	62.3	19.6	31.5%	31.0%	3.3	5.3%	32.0	59.8	126.5	94.5	151.7%
	M	34	58.8	19.4	33.0%	33.8%	3.3	5.7%	30.5	57.0	106.0	75.5	128.3%
protein content	all	70	1.5	0.5	31.7%	25.1%	0.1	3.8%	0.4	1.5	3.4	3.0	198.5%
	F	36	1.5	0.5	32.5%	29.8%	0.1	5.4%	0.7	1.4	3.4	2.7	179.7%
	M	34	1.5	0.5	31.3%	21.4%	0.1	5.4%	0.4	1.5	2.7	2.3	149.9%
TCA-WET	all	70	90.5	98.9	109.2%	81.4%	11.8	13.1%	8.9	52.8	471.5	462.7	511.0%
	F	36	92.0	100.7	109.5%	89.5%	16.8	18.3%	11.8	49.1	422.2	410.4	446.3%
	M	34	89.0	98.4	110.5%	90.7%	16.9	19.0%	8.9	52.8	471.5	462.7	519.6%
TCA-DRY	all	70	375.7	496.8	132.2%	95.8%	59.4	15.8%	29.6	176.1	2500.2	2470.6	657.5%
	F	36	396.4	553.0	139.5%	84.9%	92.2	23.3%	29.6	193.6	2500.2	2470.6	623.3%
	M	34	353.9	436.8	123.4%	98.0%	74.9	21.2%	31.3	169.4	1888.0	1856.6	524.6%
TCA-PROT	all	70	14,170.7	19,447.4	137.2%	84.6%	2324.4	16.4%	1164.9	6937.3	95,429.7	94,264.7	665.2%
	F	36	15,188.0	21,919.7	144.3%	81.6%	3653.3	24.1%	1212.7	6834.7	95,429.7	94,217.0	620.3%
	M	34	13,093.7	16,699.3	127.5%	84.4%	2863.9	21.9%	1164.9	8380.5	86,886.6	85,721.7	654.7%
GCA-WET	all	70	338.6	381.6	112.7%	90.1%	45.6	13.5%	39.8	187.2	2042.1	2002.3	591.3%
	F	36	340.1	418.5	123.1%	90.8%	69.8	20.5%	47.8	134.9	2042.1	1994.3	586.4%
	M	34	337.0	344.4	102.2%	80.6%	59.1	17.5%	39.8	226.3	1625.7	1585.9	470.6%
GCA-DRY	all	70	1398.7	1717.9	122.8%	106.3%	205.3	14.7%	124.1	743.2	7294.8	7170.7	512.7%
	F	36	1426.9	1933.7	135.5%	107.1%	322.3	22.6%	124.1	476.2	6939.9	6815.8	477.7%
	M	34	1368.8	1484.3	108.4%	102.2%	254.6	18.6%	151.8	907.0	7294.8	7143.0	521.8%
GCA-PROT	all	70	49,872.4	55,690.5	111.7%	100.9%	6656.3	13.3%	5509.7	25,135.0	307,868.4	302,358.7	606.3%
	F	36	52,192.1	67,642.6	129.6%	88.7%	11,273.8	21.6%	5509.7	21,550.9	307,868.4	302,358.7	579.3%
	M	34	47,416.3	40,248.0	84.9%	104.9%	6902.5	14.6%	5642.8	30,267.6	143,108.8	137,466.0	289.9%
CDCA-WET	all	70	183.0	138.0	75.4%	67.3%	16.5	9.0%	40.9	128.8	695.4	654.5	357.6%
	F	36	186.9	140.5	75.2%	59.9%	23.4	12.5%	49.1	133.4	695.4	646.4	345.8%
	M	34	178.9	137.4	76.8%	63.6%	23.6	13.2%	40.9	124.9	600.6	559.6	312.8%
CDCA-DRY	all	70	730.3	841.9	115.3%	66.6%	100.6	13.8%	126.7	441.1	5742.9	5616.2	769.0%
	F	36	704.3	695.1	98.7%	60.0%	115.8	16.4%	141.7	443.5	3953.4	3811.7	541.2%
	M	34	757.9	983.9	129.8%	71.4%	168.7	22.3%	126.7	436.5	5742.9	5616.2	741.0%
CDCA-PROT	all	70	30,616.0	40,125.2	131.1%	57.7%	4795.9	15.7%	3658.5	18,491.4	286,597.9	282,939.4	924.2%
	F	36	28,527.9	26,027.4	91.2%	53.6%	4337.9	15.2%	6363.4	18,086.4	106,653.1	100,289.7	351.5%
	M	34	32,827.0	51,363.1	156.5%	62.0%	8808.7	26.8%	3658.5	19,085.9	286,597.9	282,939.4	861.9%
GCDCA-WET	all	70	232.0	227.0	97.9%	85.2%	27.1	11.7%	27.0	143.0	1004.7	977.8	421.5%
	F	36	252.5	279.2	110.6%	91.5%	46.5	18.4%	27.0	119.2	1004.7	977.8	387.2%
	M	34	210.2	155.3	73.9%	80.0%	26.6	12.7%	33.9	157.5	560.3	526.4	250.4%
GCDCA-DRY	all	70	984.5	1153.4	117.2%	97.3%	137.9	14.0%	69.9	496.4	4992.6	4922.6	500.0%
	F	36	1015.5	1221.7	120.3%	94.6%	203.6	20.0%	69.9	379.6	4350.5	4280.5	421.5%
	M	34	951.6	1093.8	114.9%	91.5%	187.6	19.7%	119.8	543.2	4992.6	4872.8	512.1%
GCDCA-PROT	all	70	36,865.1	46,339.9	125.7%	93.0%	5538.7	15.0%	3425.2	18,433.4	241,160.4	237,735.1	644.9%
	F	36	41,618.9	59,103.5	142.0%	97.0%	9850.6	23.7%	3425.2	16,806.0	241,160.4	237,735.1	571.2%
	M	34	31,831.6	27,097.8	85.1%	84.4%	4647.2	14.6%	4451.4	22,461.0	119,572.2	115,120.7	361.7%
TCDCA-WET	all	70	111.4	119.4	107.2%	98.5%	14.3	12.8%	12.7	60.3	602.0	589.3	528.9%
	F	36	126.5	148.6	117.5%	105.4%	24.8	19.6%	12.8	56.5	602.0	589.2	465.6%
	M	34	95.4	76.6	80.3%	70.4%	13.1	13.8%	12.7	63.5	312.3	299.5	314.1%
TCDCA-DRY	all	70	479.4	638.0	133.1%	93.0%	76.3	15.9%	36.0	223.6	3564.8	3528.7	736.1%
	F	36	542.6	778.5	143.5%	117.5%	129.8	23.9%	36.0	198.4	3564.8	3528.7	650.3%
	M	34	412.5	446.5	108.3%	85.4%	76.6	18.6%	45.1	237.1	1720.4	1675.3	406.2%
TCDCA-PROT	all	70	18,045.2	26,240.0	145.4%	97.3%	3136.3	17.4%	1675.2	8971.4	155,139.8	153,464.6	850.4%
	F	36	21,670.6	34,166.8	157.7%	99.6%	5694.5	26.3%	1717.5	8573.3	155,139.8	153,422.3	708.0%
	M	34	14,206.7	13,116.0	92.3%	89.8%	2249.4	15.8%	1675.2	9322.3	57,540.5	55,865.4	393.2%

### 2.3. Total Bile Acid Concentration and Ratios of Individual Bile Acids

We evaluated the influences of the three normalization approaches on bile acid distributions defined by cofounders such as sex. For the total bile acid concentrations, wet weight shows the narrowest distribution, however, different normalization approaches result in individual bile acid subgroup distribution, and can, therefore influence a statistical evaluation of the data set (see Table 1 and Figure S2). Different normalization approaches do not influence the ratio of two bile acid concentrations within the same individual. Therefore, ratios of individual bile acid concentrations, their distribution, and their range, obtained from the same analytical run, are not influenced by different normalization approaches (Figure S3).

**Table 2:** Coefficient of determination (R^2)^ for the Pearson correlations of fecal taurocholic acid (TCA), glycocholic acid (GCA), chenodeoxycholic acid (CDCA), glycochenodeoxycholic acid (GCDCA) normalized by wet weight (WET), dry weight (DRY), and protein content (PROT).

		Dry Weight	Protein Content
wet weight	Pearson Corr.	0.282	−0.061
	*p*-value	0.018	0.617
dry weight	Pearson Corr.		−0.080
	*p*-value		0.511
		TCA-PROT	GCA-WET
TCA-WET	Pearson Corr.	0.934	0.888
	*p*-value	4.9 × 10^−32^	1.4 × 10^−24^
TCA-DRY	Pearson Corr.		0.871
	*p*-value		1.1 × 10^−22^
		GCA-DRY	GCA-PROT
GCA-WET	Pearson Corr.	0.889	0.844
	*p*-value	4.3 × 10^−25^	4.7 × 10^−20^
GCA-DRY	Pearson Corr.		0.881
	*p*-value		7.9 × 10^−24^
		CDCA-DRY	CDCA-PROT
CDCA-WET	Pearson Corr.	0.779	0.772
	*p*-value	2.0 × 10^−15^	5.1 × 10^−15^
CDCA-DRY	Pearson Corr.		0.574
	*p*-value		2.0 × 10^−7^
		GCDCA-DRY	GCDCA-PROT
GCDCA-WET	Pearson Corr.	0.888	0.864
	*p*-value	1.2 × 10^−24^	6.1 × 10^−22^
GCDCA-DRY	Pearson Corr.		0.855
	*p*-value		4.3 × 10^−21^
		TCDCA-DRY	TCDCA-PROT
TCDCA-WET	Pearson Corr.	0.931	0.885
	*p*-value	1.4 × 10^−31^	3.3 × 10^−24^
TCDCA-DRY	Pearson Corr.		0.846
	*p*-value		3.1 × 10^−20^

## 3. Discussion

Reliable bile acid concentrations in human feces are of fundamental interest to investigate the influence of diet and gut microbiome on human health. Feces is a challenging matrix for lipid analysis due to its highly heterogenic composition. Here we analyzed the impact of three different normalization approaches, namely wet weight, dry weight, and protein weight, on the concentrations of five bile acids. We wanted to identify the approach that shows the narrowest distribution of values for a better identification of significant differences in various biological conditions.

Our data reveal differences in wet weight and dry weight normalized fecal bile acids. Dry weight normalized bile acid concentrations show a broader spread of bile acid concentrations. However, both dry and wet weights show a significant but weak correlation. Wet weight shows also the lowest coefficient of variation of the weights, considering the fact that the water content of the samples ranges from 47–91% (see Figure 2). Four of five bile acid concentrations normalized by wet weight show the narrowest distribution. Surprisingly, bile acid concentrations normalized by wet and dry weight approaches reveal significant correlations. Protein weights are very different from the other two approaches. It has a broader distribution of weights and no significant correlation with the wet and dry weights. However, as shown in Table 2, most bile acid concentrations normalized by the three different approaches have strong, statistically highly significant correlations. These observations suggest that lipid concentrations normalized with these three different approaches may lead to a comparable overall result.

The findings are very different when it comes to bile acid concentrations in individual samples normalized by the three different approaches. Each sample has an individual composition of water, solid particles, and protein content; therefore, a sample that has high lipid concentrations normalized by wet weight might show an average or low concentration when normalized by dry weight. Each sample has an individual lipid value depending on the normalization approach used. Consequently, the identification of extreme concentrations or individuals as well as the direct comparison of individuals are highly dependent on the normalization approach (as shown in Figure 3). The same observation has been made if the data set is grouped by a cofounder, as here demonstrated for sex. Data distribution and individual concentration are also dependent on the normalization approach, which indicates that normalization approaches will also influence subgroup analyses.

Our data allow no conclusion if specific chemical properties of the bile acids increase the variability of fecal concentrations normalized by the three different approaches. We analyze here primary bile acids from which it is known that the hydrophilicity and electronegativity increase from free < glycine < taurine conjugates. The biological variability would influence an evaluation of the impact of specific chemical properties; therefore, this effect has not been evaluated in this study.

## 4. Materials and Methods

### 4.1. Materials

Two-milliliter Tough Tubes with caps, ceramic beads 1.4 mm and 2.8 mm, and Omni Bead Ruptor 24 were purchased from Omni International, Kennesaw, GA, USA. Pipette tips (200 µL Genomic LR (orifice diameter 1.5) low retention) were purchased from VWR International, Radnor, PA, USA. Data were integrated with Lab Solutions 5.97 Shimadzu Corporation, Kyoto, Japan, Lab Solutions Insight LCMS. Pierce™ BCA Protein Kit was obtained by Thermo Scientific, Waltham, MA, USA. LC-MS grade isopropanol, methanol, and acetonitrile were obtained from Fischer Chemicals, Pittsburgh, PA, USA. Taurocholic acid, glycocholic acid, chenodeoxycholic acid, glycochenodeoxycholic acid, taurochenodeoxycholic acid, and their corresponding deuterium-labeled compounds, as well as hydrochloric acid 37%, ammonium formate, and formic acid were purchased from Sigma-Aldrich, Burlington, MA, USA.

### 4.2. Equipment

Shimadzu LCMS-8060, Nexera X2 LC-30AD Liquid chromatograph, Nexera X2 SIL-30AC, CTO-20AC Prominence column, and oven were obtained from Shimadzu Cooperation, Kyoto, Japan.

### 4.3. Procedure

Human feces samples were acquired as part of a human study investigating the fitness change in experienced athletes. The study was approved by the National University of Singapore Institutional Review Board (NUS-IRB H-20-035). Samples were collected at three different time points from 42 humans aged between 38 and 61 years. From this cohort, 70 feces samples were randomly selected for subgroup analysis of bile acid concentration.

### 4.4. Faeces Preparation for Bile Acid Analysis

Sample preparation was performed by a modified protocol of Schoett et al., 2018 [15]: Raw feces material (300–500 mg) was transferred in a 2.0 mL homogenization tube, containing homogenization beads and 1.0 mL 70% (*v*/*v*) isopropanol. Samples were homogenized in an Omni Bead Ruptor (strength 6.0, time 45 s, 3 cycles, 15 s pause). From this raw homogenate, 200 µL was transferred with a pipette (using an orifice diameter of 1.5 mm), in a pre-weighed Eppendorf tube, dried in a SpeedVac overnight, and the dry weight of the sample was calculated. Subsequently, the raw extract was diluted to 2 mg/mL dry weight and 200 µL of this diluted solution was used for bile acid analysis

Bile acids were extracted for analysis by a modified protocol of Krautbauer et al., 2018 [16]. To the 200 µL diluted feces sample, 100 µL deuterated internal standard (IS in methanol), 1 mL of acetonitrile, and 30 µL of 1 mol/L HCl were added and the sample was vortexed for 1 min. Bile acids were extracted for one hour at room temperature. Subsequently, the samples were spun down in a centrifuge at 10,000 rpm, 4 °C, 15 min, and 1 mL of the upper phase was dried in a SpeedVac. After drying, the samples were reconstituted in 100 µL methanol:water (1:1 *v*:*v*). Samples were analyzed on a Shimadzu LCMS-8060, Nexera X2 LC-30AD Liquid chromatograph system in MRM negative mode. Bile acids were quantified with their corresponding deuterated standards against a matrix-free external calibration. The conjugated and free bile acids taurocholic acid (TCA 514.3→514.3; d5-TCA 518.3→124.0), glycocholic acid (GCA 464.2→74.0; d4-GCA 468.2→124.0), chenodeoxycholic acid (CDCA 391.2→391.2; d4-CDCA 395.1→395.1), glycochenodeoxycholic acid (GCDCA 448.3→74.0; d4-GCDCA 452.2→74.0), taurochenodeoxycholic acid (TCDCA 498.3→124.05; D4-TCDCA 502.4→124.0), and their deuterated internal standards were quantified with the listed transitions against a matrix-free external calibration dilution series.

### 4.5. Determination of Dry Weight, Wet Weight, and Protein Concentration

The dry weight of the diluted feces solution was determined as described above. Two hundred microliters of diluted feces solution was transferred in a pre-balanced Eppendorf tube and dried in a SpeedVac. The dry weight was determined on an analytical balance. The total amount of feces sample transferred into the homogenization tube is weighted. Moreover, the combined weight of this feces sample and the isopropanol was determined. For determination of the wet weight, the sample weight (feces and 70% isopropanol) of dried 200 µL raw feces homogenate was determined. This weight of 200 µL raw feces homogenate multiplied by the factor of wet feces within the sample is considered the wet weight of the sample. Protein concentrations were determined with a commercial Pierce™ BCA Protein kit assay. Five microliters of the diluted feces solution was used as sample for protein determination. The standard protocol of the assay kit was used for protein concentrations. Concentrations were determined in three independent technical replicates.

### 4.6. Statistical Analyses

Data were tested for normal and gaussian distribution. *p*-value < 0.05 were considered statistically significant. All statistical tests were performed with OriginPro19b (OriginLab Corporation, Northampton, MA, USA) and Excel (Microsoft Office 2019, Microsoft Corporation, Redmond, WA, USA).

## 5. Conclusions

In summary, we investigated the effects of sample wet weight, dry weight, and protein amount normalization for quantitative analysis of bile acids in feces. As expected, these three approaches result in different absolute concentrations. Bile acid concentrations normalized by these normalization approaches showed strong correlations.

Our data suggest that normalization with feces wet weight, due to the narrowest distribution of individual sample weights and normalized bile acid concentrations, is the favorable approach among the three approaches tested. Moreover, it is the most easily applicable approach. This approach may be useful in biomarker studies and while investigating the extreme values within a cohort of samples. If data are to be compared between the three approaches, the calculation of a ratio of two lipids obtained from the same analytical approach offers a good possibility. The normalization approaches tested here should be evaluated for their applicability in global as well as targeted metabolomics and lipidomics studies.

## Data Availability

Not applicable.

## Supplementary Materials

The following supporting information can be downloaded at: https://www.mdpi.com/article/10.3390/metabo12080723/s1, Figure S1: Concentrations of the five bile acids TCA (A), GCA (B), CDCA (C), GCDCA (D), and TCDCA (E) normalized by wet weight, dry weight, and protein weight in 70 human feces samples from 42 volunteers. Figure S2: Shows the individual distribution of TCA (A), GCA (B), CDCA (C), GCDCA (D), and TCDCA (E) concentrations normalized by wet weight, dry weight, and protein content. Samples are determined by a subgroup analysis of male (M) and female (F) sex. Figure S3: This figure shows the ratio of TCA/GCA (A), CDCA/GCDCA (B), and GCDCA/TCDCA (C) normalized by wet weight, dry weight, and protein content.
