# Supplementary material for: Evaluation of Normalization Approaches for Quantitative Analysis of Bile Acids in Human Feces
Source: Metabolites. 2022 Aug 5;12(8):723. doi: 10.3390/metabo12080723 (PMC9416035; doi:10.3390/metabo12080723)
Supplement: Supplementary file 1 [file metabolites-12-00723-s001.zip › metabolites-1750011-supplementary.pdf]

metabolites

MDPI

Evaluation of Normalization Approaches for Quantitative Analysis of Bile Acids in Human Feces

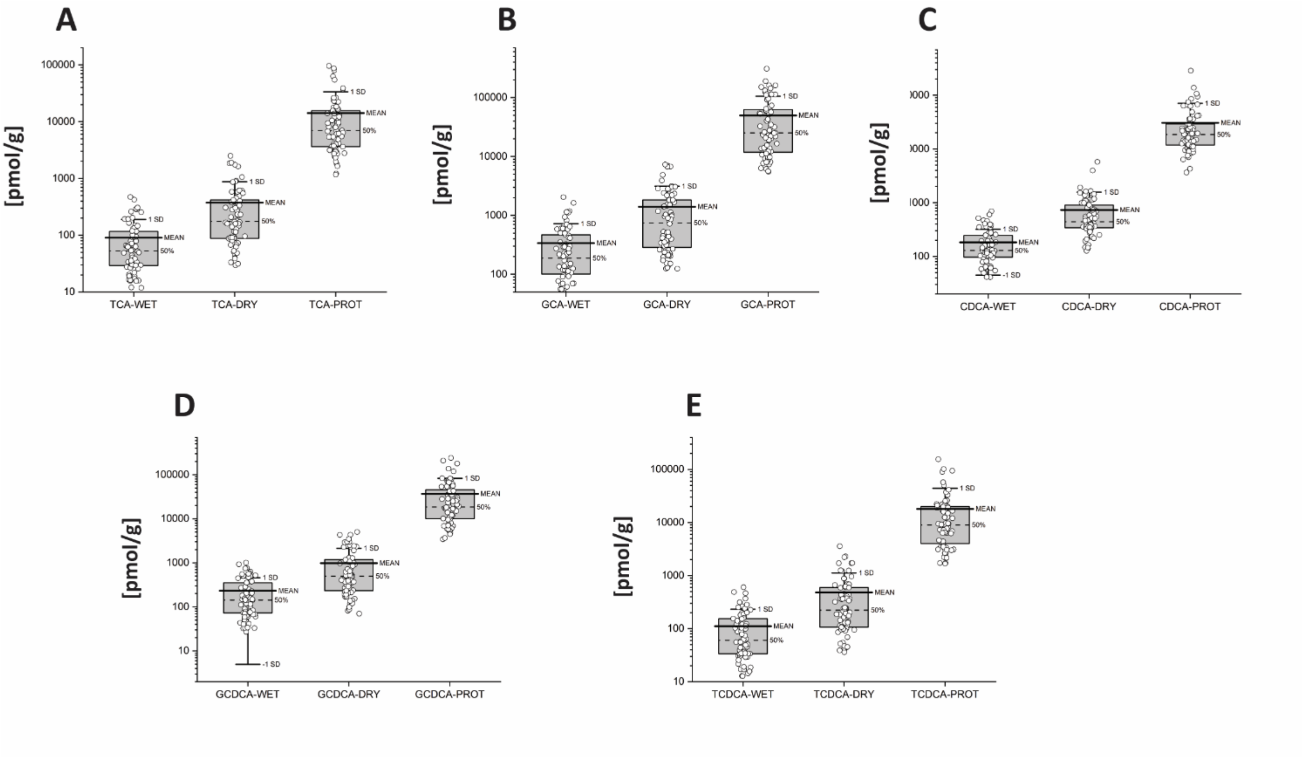

Supplementary Figure S1. Concentrations of the five bile acids TCA (A), GCA (B), CDCA (C), GCDCA (D) and TCDCA (E) normalized by wet weight, dry weight and protein weight in 70 human faeces samples from 42 volunteers.

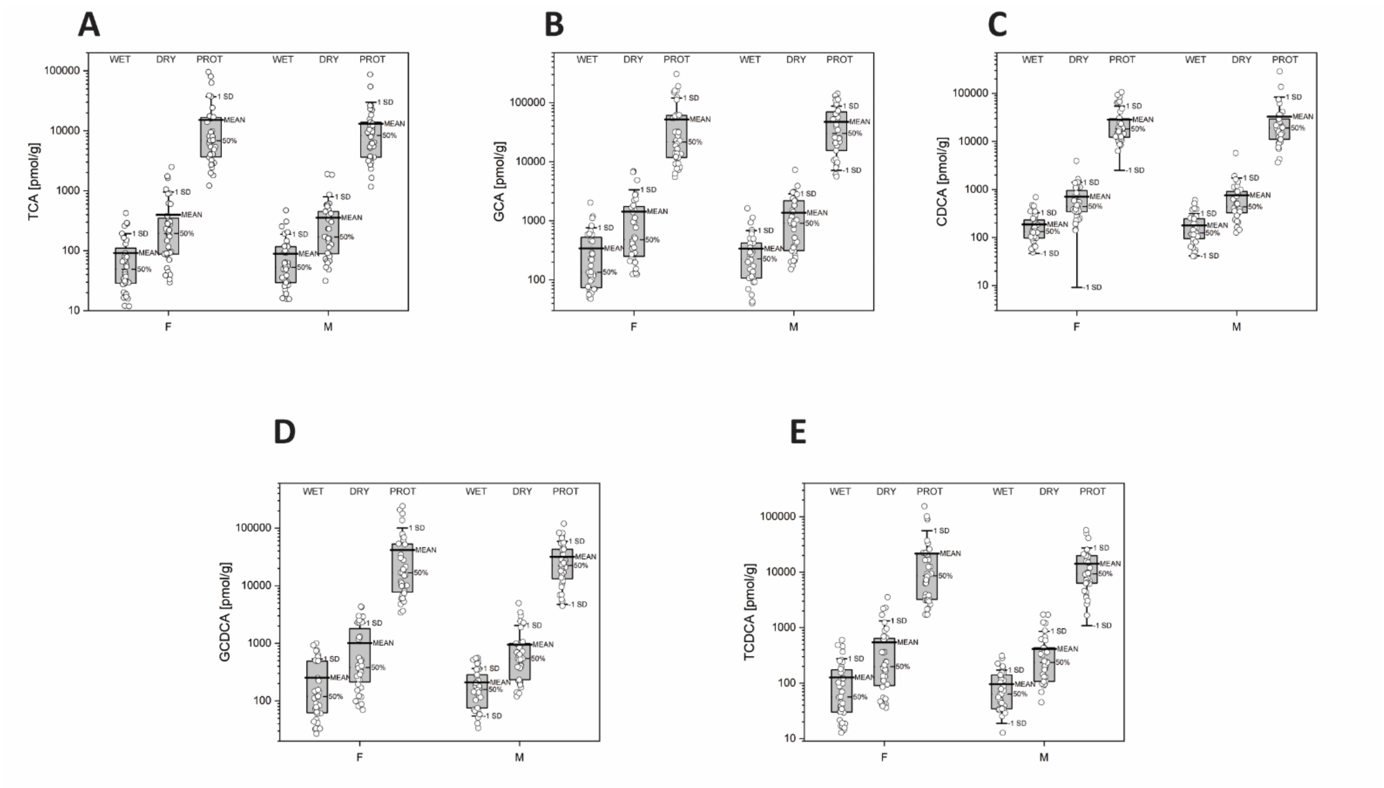

Supplementary Figure S2. Shows the individual distribution of TCA (A), GCA (B), CDCA (C), GCDCA (D) and TCDCA (E) concentrations normalized by wet weight, dry weight, and protein content. Samples are determined by a subgroup analysis of male (M) and female (F) sex.

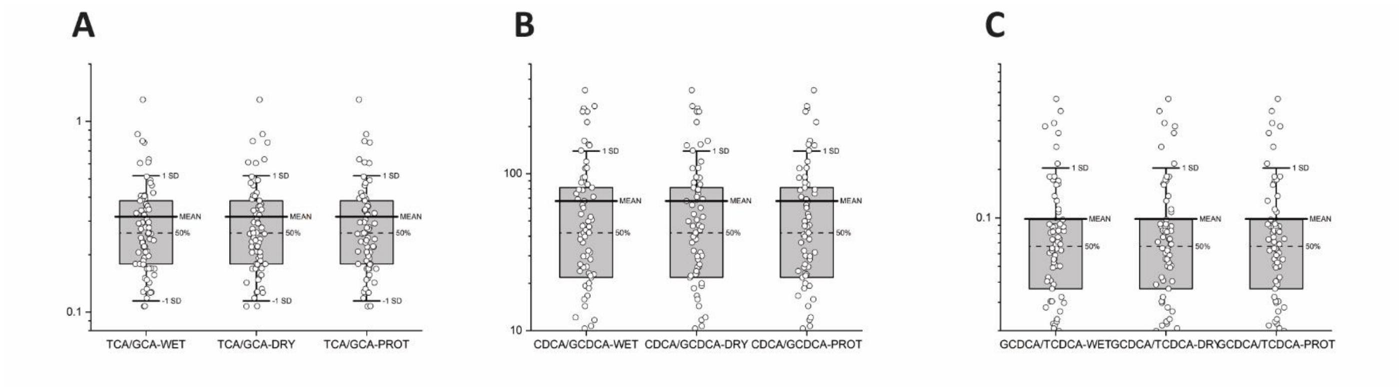

Supplementary Figure S3. Supplemental figure 3 shows the ratio of TCA/GCA (A), CDCA/GCDCA (B) and GCDCA/TCDCA (C) normalized by wet weight, dry weight, and protein content.
